# Linking Lipid Metabolism and Immune Function: New Insights into Chronic Respiratory Diseases

**DOI:** 10.3390/pathophysiology32020026

**Published:** 2025-06-06

**Authors:** Stanislav Kotlyarov

**Affiliations:** Department of Nursing, Ryazan State Medical University, 390026 Ryazan, Russia; skmr1@yandex.ru

**Keywords:** metabolism, molecular mechanisms, respiratory diseases, immunometabolism, surfactant, phospholipids, eicosanoids, pro-resolving lipid mediators

## Abstract

Lipids play important roles in maintaining pulmonary structure, performing physiological functions and controlling the immune status of the lung. There is increasing evidence that lipid metabolism and immune activity are closely linked and that dysfunction in lipid metabolism contributes to the development and progression of chronic respiratory diseases such as COPD and asthma. These diseases are characterized by metabolic and immune dysregulation, with lipid mediators playing a key role in both the development and resolution of inflammation. In this regard, lipid metabolic pathways are attracting increasing attention as promising targets for biomarker detection and therapeutic intervention.

## 1. Introduction

Respiratory diseases remain an important medical problem that is not diminishing despite numerous advances in clinical medicine and pharmacology. Indeed, acute respiratory diseases are one of the leading causes of seeking medical care, and chronic respiratory diseases remain among the leaders in terms of hospitalization and mortality. Chronic obstructive pulmonary disease (COPD), for example, is among the leading causes of death in the world, ranking third or fourth according to different data [1,2,3,4]. Both COPD and asthma also severely affect the quality of life of patients and carry a heavy economic and social burden [5,6,7,8]. Pulmonary fibrosis is another important problem, the importance of which has increased significantly in recent years, as it leads to a progressive decline in pulmonary function and, consequently, a reduced quality of life and a worse prognosis. These data indicate that the problem of respiratory diseases is still not fully solved and new research is needed to better understand the nature of these diseases and the mechanisms of their development. Advances in the study of the molecular biology of the lung have greatly improved our understanding of the pathogenesis of many respiratory diseases, which often involves impaired immune and metabolic mechanisms. There is growing evidence that immune and metabolic mechanisms are cross-linked, both at the cellular and whole organism level. The concept of cellular immunometabolism is a recognized model that demonstrates the importance of metabolic switching for the immune function of cells such as macrophages [9,10]. In addition, lipid transport mechanisms, besides ensuring lipid balance, can be utilized for immune functions. For example, reverse cholesterol transport can be used not only to remove excess cholesterol from macrophages but also to remove bacterial lipopolysaccharide (LPS) from them and transport it to the liver for utilization [11].

Lipids are characterized by great structural diversity and play a central role in lung physiology and pathology. Disturbances in the composition of lipids, including the production of lipid mediators of inflammation, are part of the mechanisms of development of chronic respiratory diseases, their extrapulmonary manifestations, and comorbid diseases [12,13,14,15,16].

Thus, the aim of the current review is to discuss the role of lipids and the place of lipid abnormalities in the mechanisms of chronic respiratory diseases.

## 2. Biological Significance of Lipids for Lung Function

### 2.1. The Lipid Landscape of the Lung

The lungs are an organ with a unique lipid biology due to their complex function of gas exchange and immune defense of the body [17]. Indeed, a huge amount of air, which contains both inorganic and organic substances as well as pathogens, passes through the lungs daily throughout life. This requires a complexly organized immune system that includes cells of the innate and adaptive immune system as well as non-immune cells, such as the airway epithelium, which also perform several immune functions.

**Figure 1:**
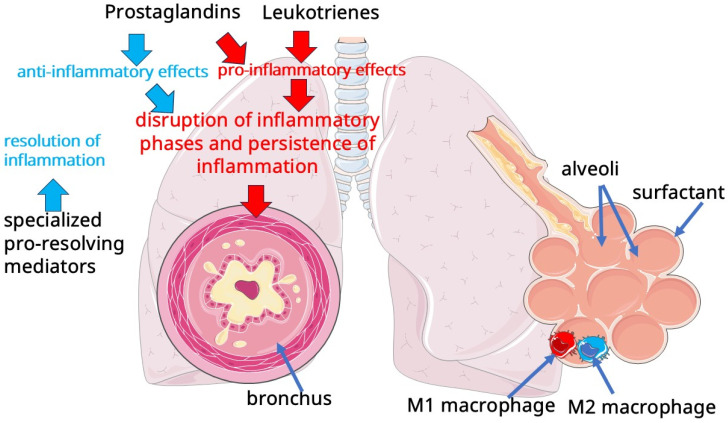
Schematic of the involvement of lipids and lipid mediators in inflammation in chronic respiratory diseases. Note: large arrows in blue color mean anti-inflammatory effect, red color pro-inflammatory effect. The artwork used in this figure was adapted from Servier Medical Art (https://smart.servier.com/, accessed on 24 May 2025), licensed under CC BY 4.0 (https://creativecommons.org/licenses/by/4.0/, accessed on 24 May 2025).

The alveolar region is represented by type I and type II alveolar epithelial cells. Alveolar epithelial type II cells (AEC2), named more than four decades ago by R.J. Mason and M.C. Williams as “defender of the alveolus”, play an important role in the lung, which is the production of pulmonary surfactant, and are also involved in some immune mechanisms (Figure 1) [18,19]. Pulmonary surfactant consists of a mixture of lipids and proteins and forms a biomolecular layer that is essential to prevent alveolar collapse and adequate gas exchange. The lipid part of the surfactant is mainly represented by phospholipids, including 80–85% phosphatidylcholine (PC) and to a lesser extent (10%) phosphatidylglycerol (PG). Most of the PC (40–60%) is represented by unsaturated dipalmitoylphosphatidylcholine [20]. Surfactant is necessary for regulation of surface tension of alveoli, which provides effective ventilation, preventing alveolar collapse, and affects bronchial patency and regulates the balance of fluid in the airways. The surface tension maintained by surfactant acts as an additional force to direct fluid flow across the air–blood barrier, which prevents the formation of alveolar edema. In addition to its involvement in maintaining the biophysical properties of the alveoli, surfactant exhibits immunomodulatory activity that includes suppression of cytokine secretion and activation of transcription factors [21,22,23,24]. 

Phosphatidylglycerol (PG) and phosphatidylinositol (PI) present in the alveolar regions of the lungs have significant immunomodulatory properties [25,26]. They inhibit inflammatory responses triggered by several Toll-like receptors (TLRs) [26]. It has been shown, for example, that PI and palmitoyl-oleoyl-phosphatidylglycerol (POPG), one of the dominant molecular species of PG, inhibit inflammatory responses induced by different Toll-like receptors by interacting with subsets of multi-protein-coupled receptor components [26]. These lipids also exert antiviral effects against respiratory viruses by inhibiting virus binding to host cells [25,26]. 

Surfactant lipids, including phosphatidylcholines (PCs), such as 1-palmitoyl-2-oleoyl-sn-glycero-3-phosphocholine (POPC), have been shown to modulate the activity of alveolar macrophages and enhance the production of inflammatory mediators after stimulation with lipopolysaccharide (LPS) [27]. POPC reacts with ozone to produce 1-palmitoyl-2-(9-oxo-nonanoyl)-sn-glycero-3-phosphocholine (PONPC). Alveolar macrophages preincubated with POPC or PONPC showed an enhanced response after lipopolysaccharide (LPS) stimulation and increased nitric oxide and cytokine production [27]. In addition, lipid components of surfactant, such as dipalmitoylphosphatidylcholine (DPPC), can modulate inflammatory responses in the lung by suppressing interleukin-8 (IL-8) expression in A549 lung epithelial cells. The immunomodulatory effect of surfactant lipids is to suppress translocation of Toll-like receptor 4 (TLR4) to lipid raft membrane domains, which is a crucial mechanism for the regulation of immune responses [28].

In addition to lipids, surfactant is composed of several proteins, including surfactant protein A (SP-A) and surfactant protein D (SP-D) from the C-type lectin family. SP-A and SP-D bind to pathogens, promoting their opsonization and removal by phagocytes such as macrophages. SP-A and SP-D interact with various immune cells including macrophages, dendritic cells, and T lymphocytes, modulating their functions and enhancing pathogen destruction [29,30,31]. SP-A binds to dipalmitoylphosphatidylcholine (DPPC), a major lipid component of surfactant, while SP-D binds to phosphatidylinositol (PI) [32]. The affinity of SP-A for DPPC is crucial for its role in surfactant structure and body defense [32,33]. Surfactant protein D has also been found to suppress lipid-laden foamy macrophages and lung inflammation in COPD. SP-D increases the expression of genes involved in combating oxidative stress and lipid metabolism disorders induced by cigarette smoke and oxLDL in bone-marrow-derived macrophages (BMDMs) [34].

Cholesterol accounts for a much smaller proportion compared to phospholipids in surfactant: approximately 8–10% by weight or 14–20 mol% of alveolar surfactant in human and placental mammalian lungs. The cholesterol content of surfactant in animals decreased evolutionarily from fish to placental mammals, corresponding to the transition from cold-bloodedness to warm-bloodedness and evolutionary improvement of lungs. The decrease in cholesterol content in surfactant corresponded to an increase in phospholipid content [35]. Indeed, the relative proportion of cholesterol in surfactant increases rapidly when body temperature decreases in a number of animals when they go into torpor. In this regard, cholesterol is considered a protosurfactant in the early lungs of amphibians and reptiles, where it also probably serves an antioxidant function, whereas phospholipids are an evolutionarily newer acquisition [36].

The composition of surfactant can change significantly in various pathological conditions. Smoking leads to impaired phospholipid composition of surfactant, which may impair its function [37,38,39]. In a mouse model of COPD produced by exposure to tobacco smoke, it was shown that there was a correlation between lung function and the content of phospholipids, cholesterol, and sphingomyelin in bronchoalveolar lavage. The greatest correlation of pulmonary function was with phosphatidylcholine, which reduces surface tension, and phosphatidylglycerol, which has antimicrobial properties [40]. In addition, pathways involved in phospholipid metabolism and degradation, including genes encoding phospholipases, were activated in human and mouse lungs by smoking [41]. Exposure of mice to cigarette smoke for 6 months resulted in the accumulation of oxidized phospholipids in bronchoalveolar lavage fluid. The accumulation of oxidized phospholipids suppressed the phagocytic function of alveolar macrophages in these mice, consistent with impaired bacterial phagocytosis and bacterial excretion by alveolar macrophages [42]. Lipid peroxidation and abnormalities in lipid composition are well known in COPD, which develops with a long history of smoking [43,44]. Phagocytosis disorders are part of the pathogenesis of COPD, which contributes to bacterial colonization of the bronchi and disease progression [45].

Surfactant also has some immune functions. The involvement of pulmonary surfactant in defense mechanisms is due to the fact that it, for example, contains homeostatic and antimicrobial hydrolases that can affect the cell membrane of *Mycobacterium tuberculosis*, alter the intracellular transport of *M. tuberculosis*, and induce a protective pro-inflammatory response to infection [46]. Age-related dysfunction of soluble components of innate immunity in human alveolar lining fluid has been shown to result in accelerated growth of *M. tuberculosis* in human alveolar macrophages [47].

AEC2 cells regulate lipid metabolism to maintain surfactant synthesis. The formation of fatty acids, which are used to synthesize phospholipid esters, is carried out by the enzyme fatty acid synthase (FASN) [48,49]. This enzyme in the lung is expressed predominantly in AEC2 cells and its expression is decreased in COPD. Loss of FASN in AEC2 cells has been shown to alter the lipid profile of the lung, which may accelerate the development of emphysema. Exposure to cigarette smoke in an experiment enhanced lipid biogenesis in AEC2 cells and altered the composition of surfactant phospholipids. This is consistent with a decrease in FASN gene and protein expression in lung homogenates from mice exposed to smoke for 6 months [48].

Lysophosphatidylcholine acyltransferase 1 (LPCAT1) is an enzyme that catalyzes surfactant lipid biosynthesis and is expressed in AEC2 cells. LPCAT1 is involved in the conversion of phosphatidylcholine to dipalmitoylphosphatidylcholine, which is responsible for reducing the surface tension of surfactant [50]. The decrease in LPCAT1 levels induced by cigarette smoke may contribute to the exacerbation of pulmonary emphysema by increasing the susceptibility of alveolar epithelial cells to apoptosis [51]. In another study, lysophosphatidylcholine acyltransferase levels were shown to predict severity and prognosis in patients with community-acquired pneumonia, which may also reflect the degree of impaired surfactant formation [52]. 

In addition to AEC2 cells, alveolar macrophages also play a crucial role in the regulation of surfactant homeostasis. These cells are involved in the excretion and degradation of pulmonary surfactant, which is controlled by several mechanisms [38]. 

Excess external cholesterol has been shown to be associated with inflammation in airway epithelium [53]. It was found that external cholesterol is involved in the regulation of intracellular cholesterol transport and accumulation, regulation of StAR Related Lipid Transfer Domain Containing 3 (STARD3)–Mitofusin 2 (MFN2) pathways and mitochondrial dysfunction in bronchial epithelium. Cholesterol accumulation in airway epithelial cells is accompanied by suppression of the sterol-regulatory element-binding protein 2 (SREBP2)/low-density lipoprotein receptor (LDLR) pathway and the cholesterol biosynthesis pathway. Through these mechanisms, external cholesterol altered the sensitivity of airway epithelium to inflammation in response to cigarette smoke extract (CSE) [53].

Increased cholesterol synthesis and decreased phosphotidylcholine levels have been found to increase susceptibility to emphysema [48]. In an experiment, Apoe−/− mice fed a Western diet exhibited severe systemic hypercholesterolemia, which corresponded to lung inflammation and emphysema development via the TLR4/inflammation/MMP cascade [54]. In contrast, statins have been shown to exert anti-inflammatory effects by reducing cytokine production through inhibition of the mevalonin cascade with subsequent activation of RhoA in the lungs [55]. Simvastatin administration prevents smoking-induced damage to the airway epithelium [56]. Simvastatin modulates gene expression of several pro-inflammatory cytokines and chemokines induced by IL-13 (eotaxin-1; MCP-1,-2,-3; and osteopontin (SPP1)) in primary mouse tracheal epithelial cells [57]. When administered intratracheally, pravastatin reduced metaplasia/hyperplasia of bocaloid cells, but no statistically significant anti-inflammatory effect was found, except for a decrease in the levels of some cytokines such as TNF-α [58].

Thus, the lungs have a complexly organized and evolutionarily shaped lipid composition that is essential for their respiratory and immune functions. Disorders of lipid composition and lipid metabolism play an important role in the development and progression of chronic respiratory diseases.

### 2.2. The Importance of Lipids for Macrophage Function

#### 2.2.1. Concept of Cellular Immunometabolism

Alveolar macrophages are an important population of cells in the lungs that professionally perform immune functions. The number of these cells is significantly increased in the lungs in COPD, and they are both of pulmonary origin, being part of the tissue macrophage population, and differentiated from recruited blood monocytes [59,60,61].

The concept of cellular immunometabolism, which suggests a link between the function of cells in immunity and the peculiarities of their metabolism, is of growing interest to researchers. Indeed, macrophages can differentially participate in inflammation, contributing to both its activation and resolution. According to this concept, which has been exemplified in murine macrophages, these cells can perform pro-inflammatory functions (M1 macrophage polarization) and anti-inflammatory or reparative functions (M2 macrophage polarization). Such polarization corresponds to the phases of inflammation, when at the initial stage its activation is required to attract new immune cells, detect and destroy the pathogen, and later the processes of inflammation resolution and tissue repair are required. It has been found that macrophage polarization occurs due to a switch in the metabolism of these cells [62,63,64]. In M2 polarization, the main sources of energy for cells are oxidative phosphorylation, which also corresponds to a normal tricarboxylic acid cycle and moderate glycolytic activity, and fatty acid oxidation. At M1 polarization, glycolysis and fatty acid synthesis are greatly enhanced, while the tricarboxylic acid cycle and oxidative phosphorylation are disrupted. The disrupted tricarboxylic acid cycle is an important source of metabolites that are involved in biosynthetic and immune processes [65,66,67]. In addition, M1 and M2 macrophages metabolize arginine differently, which was originally the basis for their subtype division. While M1 macrophages use arginine to form bactericidal nitric oxide via inducible nitric oxide synthase (iNOS), M2 macrophages metabolize it via arginase to ornithine and then proline, which are used for tissue repair after inflammation [67,68,69]. Disruption of macrophage polarization may contribute to the persistence of inflammation in smoking and COPD. In addition, dual polarization of alveolar macrophages, i.e., co-expression of M1 and M2 markers in the same alveolar macrophage, may occur in smoking and COPD and increases with increasing severity of COPD [70].

Lipid metabolism in pro-inflammatory M1 macrophages involves enhanced synthesis of fatty acids, which utilizes metabolites removed from other metabolic pathways such as glycolysis, the Krebs cycle, and the pentose phosphate pathway. Carbon atoms derived from glucose during enhanced glycolysis in LPS-activated macrophages have been shown to be preferentially incorporated into fatty acids and sterols. Citrate and fatty acids have been shown to increase in LPS-activated macrophages. In addition, cholesterol biosynthesis is increased in pro-inflammatory activated macrophages. Cholesterol biosynthesis occurs in the endoplasmic reticulum, and the source of carbon atoms for this is acetyl-CoA coming from mitochondria as part of citrate, just as in the synthesis of fatty acids. Thus, lipid biosynthesis in macrophages is linked to tricarboxylic acid cycle activity via acetyl-CoA. This metabolite serves as a precursor in the biosynthesis of major classes of lipids, including fatty acids, cholesterol, eicosanoids, and complex lipids such as glycerophospholipids and sphingolipids [66,71,72,73,74].

Thus, the involvement of macrophages in the different phases of inflammation has a complex immunometabolic basis, which is composed of basic metabolic pathways such as glycolysis, the tricarboxylic acid cycle, and oxidation or synthesis of fatty acids. Although there are some species-specific features in the described processes, the immunometabolism model is of interest as an example of the cross-talk between cellular metabolism and immunity. Changes in lipid metabolism also contribute to the immune activity of macrophages, which has implications for the course of inflammation.

The lipid profile of M1 and M2 macrophages was also found to be different: M1 macrophages contain a higher level of cholesterol esters and triacylglycerols rich in polyunsaturated fatty acids. In turn, M2 macrophages are characterized by high levels of glycerophospholipids, ester lipids, and sphingolipids. Triglycerides in this subtype of macrophages consist predominantly of saturated fatty acids. The diacylglycerol acyltransferase enzyme promotes the conversion of free fatty acids to triglycerides, which is a defense mechanism of M1 macrophages against lipotoxicity [75]. Triglyceride synthesis is essential for normal inflammatory activity of macrophages. Inhibition of triglyceride synthesis in pro-inflammatory macrophages results in reduced lipid droplets but impairs phagocytosis and disrupts the production of inflammatory mediators, including interleukin (IL)-1β, IL-6, and prostaglandin E2 (PGE2). Moreover, the addition of exogenous PGE2 is able to prevent the anti-inflammatory effects of triglyceride synthesis inhibition [76].

Immunolipidomic profiling of macrophages after their activation by lipopolysaccharide has also been shown. Pro-inflammatory and reparative phases of activation in human macrophages are associated with reprogramming of lipid metabolism. Lipopolysaccharide, acting through TLR4, induces sphingolipid synthesis in macrophages mainly during the first three hours, while their products accumulate during the resolution phase [73,77]. Sphingolipids are required for many immune functions of macrophages, including pathogen phagocytosis, lysosome function, vesicle fusion, autophagy, and antigen presentation [77,78,79,80,81]. Interestingly, the intact sphingolipid biosynthesis pathway is required by *M. tuberculosis* for its uptake by phagocytes and consequently the development of infection [82].

Interestingly, saturated fatty acids such as palmitic acid can bind directly to the TLR4/MD-2 complex, leading to dimerization and activation of the receptor [83,84]. This binding is facilitated by the hydrophobic nature of saturated fatty acids, which allows them to interact with the hydrophobic binding pocket of the MD-2 adaptor protein [83]. Saturated fatty acids promote the movement of TLR4 into lipid rafts in the plasma membrane, which is a critical step for receptor activation and subsequent inflammatory signaling [84,85,86]. This process can be slowed down by polyunsaturated fatty acids such as docosahexaenoic acid (DHA) [84,86]. Studies have shown that saturated fatty acids such as palmitic acid can stimulate TLR4-dependent signaling pathways, leading to the production of pro-inflammatory cytokines such as IL-1β and TNF-α in various cell types including macrophages and endothelial cells [83,85]. TLR4 activation by free fatty acids has been shown to contribute to chronic inflammation associated with obesity and metabolic disorders [87,88,89].

Exposure to cigarette smoke and vaping can also lead to lipid accumulation in pulmonary macrophages. This is because cigarette smoke may exert LPS-like pro-inflammatory effects, including stimulating M1 polarization of macrophages [90]. High levels of lipid-laden macrophages were found in bronchoalveolar lavage from almost all smokers and half of e-cigarette users but were not found in those who had never smoked [91]. In this regard, lipid-laden macrophages are considered as biomarkers of lung damage associated with vaping [92]. Furthermore, in people with lung damage associated with e-cigarette and vaping use-associated lung injury (EVALI) alveolar macrophages show evidence of an inflammatory phenotype, or M1 phenotype. However, patients who used e-cigarettes but did not have EVALI were found to have macrophages with an M2 phenotype, as were healthy controls [93].

Lipid-laden macrophages are found in various lung diseases such as pneumonia, severe asthma, exogenous allergic alveolitis, or cystic fibrosis, but are also frequently seen in extrapulmonary causes such as aspiration in children or in gastroesophageal reflux disease [94,95,96,97].

Tuberculosis also produces a type of macrophage filled with lipid droplets. In contrast to atherosclerotic foam cells containing cholesterol, in tuberculosis the lipids in macrophages consist predominantly of triglycerides. These triglycerides are both synthesized by macrophages and taken up by them, including by phagocytosis of apoptotic or necrotic cells [98].

#### 2.2.2. The Importance of Reverse Cholesterol Transport

As already mentioned, lipid metabolism plays an important role in the function of alveolar macrophages. Cellular accumulation of cholesterol in macrophages contributes to their pro-inflammatory activation, as well as a decrease in phagocytic activity. Cholesterol accumulation is associated with several known mechanisms, including reduced cholesterol efflux due to decreased functional activity of ATP Binding Cassette Subfamily A Member 1 (ABCA1) by smoking. Decreased expression and functional activity of ABCA1 promotes the transformation of macrophages into “foam cells”, which mediates many pro-inflammatory effects. ABCA1 through regulation of reverse cholesterol transport plays an important role not only in atherogenesis but also in respiratory diseases [99]. This is due to the ability of ABCA1 to regulate macrophage pro-inflammatory activity, through membrane cholesterol content, lipid raft stability, and associated TLR signaling pathways [100]. Reverse cholesterol transport affects the cholesterol content in the plasma membranes of macrophages, thus regulating their participation in inflammation. This is due to the fact that cholesterol is a key component of membranes and largely determines their biophysical properties. Cholesterol is involved in the formation of lipid rafts, that is, liquid-ordered fractions of the plasma membrane that serve as platforms for various signaling molecules. Membrane proteins, such as TLR4 receptors, localize in lipid rafts, and the cholesterol they contain provides the necessary spatial arrangement of the protein, including through cholesterol–protein interactions. One such domain is the amino acid cholesterol-binding domain (cholesterol recognition/interaction amino acid consensus sequence (CRAC)) [101,102,103]. The amino acid sequence of CRAC and its CARC counterpart in or near the transmembrane region of the protein may indicate the possible involvement of cholesterol in the regulation of protein function. TLR4 has multiple cholesterol binding domains (CARC-CRAC-CARC domains). In this regard, the cholesterol content in the plasma membrane may influence the pro-inflammatory function of TLR4 [104]. Due to this mechanism ABCA1 may participate in the regulation of airway inflammation in COPD.

ABCA1-mediated abnormalities of reverse cholesterol transport may be associated with the regulatory influence of LXR [105]. In patients with COPD there is an increase in liver X receptor (LXR) expression in small airway epithelium and alveolar epithelium but not in alveolar macrophages [106]. LXR activation in the lungs of COPD patients may be associated with increased levels of 25- and 27-hydroxycholesterol, which are endogenous ligands for LXR. The formation of these metabolites is associated with increased expression of cholesterol 25-hydroxylase and sterol 27-hydroxylase in alveolar macrophages and pneumocytes of COPD patients [107,108]. The concentration of cholesterol 25-hydroxylase in sputum was inversely related to the percentage of predicted forced vital capacity of the lungs and forced expiratory volume in 1 second (FEV1). Moreover, sputum 25-hydroxycholesterol concentration was significantly correlated with sputum interleukin (IL)-8 levels and neutrophil counts [108]. In addition, cholesterol-25-hydroxylase was identified as a major lipid metabolism gene in asthma and showed high expression in two mouse models of asthma [109].

This suggests that cholesterol metabolites, fatty acids, and tobacco smoke may influence LXR-dependent cholesterol efflux in lung tissue in COPD. In this case, LXR activation has an anti-inflammatory effect on the production of C-X-C motif chemokine ligand 10 (CXCL10), (C-C motif) ligand 5 (CCL5), and IL-10 by alveolar macrophages [106].

Thus, reverse cholesterol transport plays an important role in cholesterol metabolism and lung function.

### 2.3. The Role of Lipid Mediators of Inflammation

Inflammation is an important universal defense mechanism that responds to various tissue injuries. Inflammation involves both cellular and humoral mechanisms and has an initialization phase and a resolution phase, which is necessary to ensure tissue immune homeostasis. Both phases are mediated by several biological factors, including lipid mediators. Indeed, there is increasing evidence that lipids have more complex functions than simply being a source of energy or structural material for cells. Lipid mediators are actively involved in both the initiation and maintenance of inflammation and mediate the highly organized phase of inflammation resolution.

**Figure 2:**
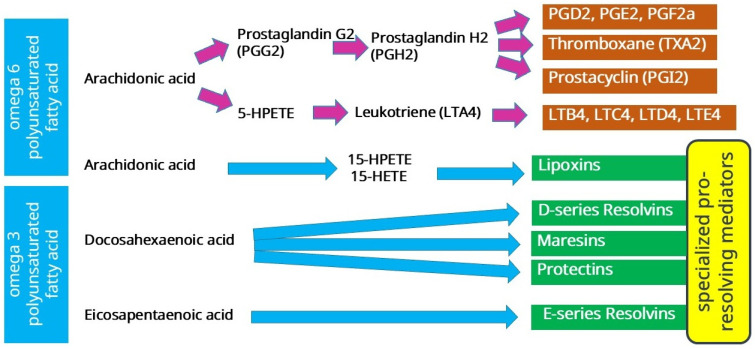
Pathways of biosynthesis of lipid mediators of inflammation.

Prostaglandins and leukotrienes are known to play an important role in inflammation. Some of them are also well known for their role as inducers of pathophysiologic processes in the airways, causing inflammation and bronchospasm [110]. The biosynthesis of these eicosanoids begins with the release of arachidonic acid (Figure 2) from membrane phospholipids by the action of phospholipase A2 (PLA2), which occurs under various physiologic and pathologic influences.

Leukotriene biosynthesis involves the enzymatic conversion of arachidonic acid under the action of 5-lipoxygenase (5-LOX) via intermediate hydroperoxyeicosatetraenoic acids (5-hydroperoxyeicosatetraenoic acid, 5-HPETE) to unstable leukotriene A4 (LTA4), which is converted to LTB4 by the enzyme LTA4 hydrolase (LTA4H) or converted to LTC4 by LTC4 synthase (LTC4S), depending on the cell type and specific enzyme expression. Specifically, leukotriene B4 (LTB4) is formed in neutrophils and monocytes by the action of LTA4 hydrolase, and leukotriene C4 synthase is expressed in eosinophils, mast cells, and platelets to produce leukotriene C4 (LTC4). LTC4 can be metabolized by γ-glutamyl transpeptidase to LTD4, which is further converted to LTE4 by dipeptidase [110,111]. These lipid mediators, i.e., LTC4, D4, and E4, are also called cysteinyl-LT (CysLT) and are known for their bronchoconstrictor properties. They also promote the recruitment and activation of cells that play a key role in the development of asthmatic inflammation, cause mucosal edema due to increased vascular permeability, promote mucus hypersecretion, and decrease mucociliary clearance [110].

Prostanoids, representing another subgroup of eicosanoid mediators, are formed as a result of oxidation of polyunsaturated fatty acids via the cyclooxygenase pathway (constitutive cyclooxygenase (COX)-1 and inducible COX-2). Their chemical structure includes a 5-carbon cyclopentane ring, which is formed by the two-step enzymatic activity of cyclooxygenases [111]. Biosynthesis of prostaglandins involves first the formation of PGG2 with the participation of cyclooxygenase isoenzymes, which is then reduced to PGH2 by peroxidase. PGH2 in turn is converted to biologically active metabolites such as PGE2, PGI2, PGD2, and PGF2a, as well as thromboxane (TXA2) by PG synthases. These eicosanoids have versatile functions due to the diverse function of their receptors [112].

The physiologic function of PGD2 varies between cell and tissue types and depends on the type of receptor to which it binds. PGD2 plays an important role in the regulation of allergic inflammation in asthma, with PGD2 levels being higher in patients with asthma than in patients with COPD [110,113]. PGD2 is also involved in the pathogenesis of cough attacks [114]. In addition, PGD2 and PGF2α promote the production of mucus glycoproteins, whereas PGE2 conversely decreases their release. Overall, PGD2 exerts many different effects on the airways relevant to asthma, including regulation of mucus production and capillary permeability [115,116,117]. PGD2 has been shown to be a potent bronchoconstrictor [118]. The combination of PGD2 and leukotriene E4 (LTE4) enhances cytokine production by TH2 cells through different mechanisms, stimulating a variety of downstream effector functions, including neutrophil migration and survival [119].

The mechanism of action of PGE2 also depends on the cell type and the receptor to which it binds. PGE2 exerts a variety of complex biological functions by binding to various prostaglandin E receptors, including EP1, EP2, EP3, and EP4 [120]. It has been shown that prostaglandin E2 and its receptors EP2 and EP4 are likely involved in the relaxant effect of pulmonary surfactant in the airways [121]. Prostaglandin E2 activates EP2 receptors, inhibiting degranulation of human pulmonary mast cells. 

As mentioned above, inflammation resolution is a complexly regulated process that normally follows the phase of inflammation activation. In COPD, persistent inflammation is characterized by disruption of the normal alternation of inflammatory phases, when both activation and resolution of inflammation occur simultaneously in the lungs. Representatives of the family of lipid mediators, called “specialized pro-resolving mediators” (SPMs), are involved in inflammation resolution [122,123]. This family includes several classes of lipid mediators, including lipoxins, resolvins, protectins, and maresins [124,125,126,127,128]. SPMs are formed enzymatically from ω-3 and ω-6 polyunsaturated fatty acids (PUFAs) such as arachidonic acid, eicosapentaenoic acid, and docosahexaenoic acid. Lipoxins are synthesized from arachidonic acid, eicosapentaenoic acid produces E-series resolvins, and docosahexaenoic acid synthesizes protectins, D-series resolvins, and maresins (Figure 2). Thus, PUFAs play an important role in the regulation of inflammation, being a substrate for the formation of lipid mediators. Moreover, arachidonic acid is used for the formation of both pro-inflammatory and anti-inflammatory mediators. The regulation of the delicate balance in the formation of pro- or anti-inflammatory lipid mediators is complex and largely unknown. What is known, however, is that lipid mediators coordinately regulate inflammation. It has been shown, for example, that the production of lipid mediators of inflammation such as leukotrienes and prostaglandins is coordinated with neutrophil recruitment. First, the chemoattractant leukotriene B4 (LTB4) promotes neutrophil recruitment, and later PGE2 promotes a switch of the biosynthesis pathway involving 5-lipoxygenase (5-LOX) from LTB4 to LXA4, resulting in reduced tissue infiltration by neutrophils [129,130,131,132,133]. Thus, lipid mediators are involved in the coordination of inflammation phase change.

The first class of SPMs identified were lipoxins, which include lipoxin A4 (LXA4), lipoxin B4 (LXB4), and their epimers: 15-epi-LXA4 and 15-epi-LXB4. Lipoxins are synthesized from arachidonic acid by the sequential action of 5-, 12-, and 15-LOX. The anti-inflammatory effects of LXA4 are multi-faceted and include regulation of immune cell chemotaxis and their interaction with epithelial cells. LXA4 has been shown to inhibit IL-8 production by leukocytes and bronchial epithelial cells, reduce neutrophil chemotaxis, and inhibit their interaction with epithelial cells. LXA4 also stimulates chemotaxis and adhesion of monocytes, increasing the uptake of apoptotic neutrophils by macrophages, which promotes the clearance of apoptotic leukocytes by macrophages in the focus of inflammation [131,134,135].

Resolvins are another class of specialized pro-resolving lipid mediators and are synthesized from ω-3 PUFAs such as eicosapentaenoic acid (EPA) and docosahexaenoic acid (DHA). The term resolvins reflects their role as a participant in the resolution of inflammation. The D-series resolvins are currently known to include RvD1, 2, 3, 4, 5, 6. These are formed from DHA, and the E series resolvins (RvE1, 2, 3, 4) are formed from EPA. In addition, epimers of these classes of resolvins are known to form when cyclooxygenase is inhibited by aspirin [136,137].

RvD1 is involved in the regulation of neutrophil migration across the endothelium and promotes neutrophil efferocytosis and M2 polarization of macrophages. Other resolvins also exert anti-inflammatory effects through regulation of neutrophils and macrophages [138,139,140,141]. RvE1, for example, reduces neutrophil migration, enhances phagocytosis of apoptotic neutrophils by macrophages, and suppresses the release of inflammatory mediators and regulates macrophage differentiation [142,143,144,145,146,147,148,149,150,151,152].

Protectins (PDs) are another family of SPMs that are synthesized from docosahexaenoic acid (DHA) and docosapentaenoic acid (DPA) [153]. PD1 acts in an anti-inflammatory manner, including inhibiting neutrophil migration, as well as causing a decrease in their production of TNF-α and IFN-γ and regulating CCR5 expression [154,155,156]. In addition, PD1 also has anti-apoptotic activity, stimulating phagocytosis and efferocytosis by macrophages that engulf apoptotic neutrophils [126,146,157,158]. 

Maresins (MaRs) are another family of SPMs. Maresins are formed in macrophages from ω-3 docosahexaenoic acid (DHA). The name of the class refers to their macrophage origin (MAcrophage RESolving INflammation) [159]. Several maresins are known, including MaR1, MaR2, MaR1-d5, MaR2-d5, and three conjugated maresins MCTR1, MCTR2, MCTR3 [160]. Maresins also exert anti-inflammatory effects. They enhance neutrophil activation, activate phagocytosis and efferocytosis of macrophages, and promote the switch of macrophage phenotype from the M1 to M2 phenotype [123,124,161,162,163,164,165,166]. Data on the possible therapeutic potential of maresins are of interest. High doses of MaR1 have been shown to effectively prevent OVA-induced inflammatory cell infiltration and excessive mucus production in lung tissues. In addition to reducing the number of inflammatory cells in bronchial lavage waters, MaR1 suppresses the expression of inflammatory factors. This action is realized through the NF-κB signaling pathway, degradation of IκBα, and expression of inflammatory genes downstream of NF-κB, such as COX-2 and ICAM-1, in a mouse model of OVA-induced asthma. The results of this study suggest that MaR1 may be useful in the treatment of asthma [167]. In another study, maresin-2 was shown to reduce inflammatory cell infiltration and oxidative stress in OVA-induced asthma in mice by suppressing NLRP3 inflammasome activation and subsequent IL-1β and IL-18 production, as well as Th2 immune response and oxidative stress [168]. These data demonstrate a potential therapeutic effect of MaR2 in a mouse model of OVA-induced asthma.

Thus, lipid mediators of inflammation and specialized pro-resolving lipid mediators are of significant clinical and research interest as a diagnostic marker and promising therapeutic target.

## 3. Clinical Significance of Lipids in Lung Diseases

### 3.1. Clinical Significance of Lipid Metabolism Disorders in COPD

COPD is associated with systemic manifestations, including disorders of lipid metabolism that contribute to the development of comorbidities. A growing body of evidence suggests that impaired lipid metabolism in the lungs is an important part of the processes that are associated with the development and progression of COPD [14,17]. Metabolic syndrome, diabetes mellitus, and atherosclerosis are frequently found in COPD patients and are some of the key causes of hospitalizations and death in these patients. Interestingly, some studies have shown a negative association of low body mass index (BMI) with prognosis in COPD. This is primarily due to the fact that low BMI is associated not only with a reduction in fat mass but also in muscle mass, which is an important metabolically active organ. Moreover, some data suggest a better prognosis in COPD patients with excess body weight. This phenomenon has been termed the “obesity paradox” and is an additional factor reinforcing the attention to lipid metabolism in COPD [169,170].

COPD is often accompanied by abnormalities of lipid metabolism, including dyslipidemia. These abnormalities include changes in LDL, high-density lipoprotein (HDL), and triglyceride (TG) levels [171,172,173]. COPD patients also have increased levels of oxidized LDL in serum compared to healthy control subjects. Moreover, elevated serum levels of oxidized LDL correlate with lung function, inflammation, and oxidative stress in COPD [174]. In addition, lipid ratio and oxidative stress level in COPD patients are closely related to the prognosis of pulmonary hypertension [175].

Lipoprotein-associated phospholipase A2 (Lp-PLA2) is a specific subtype of phospholipase A2 secreted by macrophages, T cells, and vascular endothelial cells. Approximately 20% of circulating Lp-PLA2 enzyme binds to HDL and the remaining 80% binds to atherogenic LDL. Lp-PLA2 levels were found to be significantly higher in patients with diffuse pulmonary emphysema, obstructive ventilatory dysfunction, and small airway dysfunction, suggesting a potential link between Lp-PLA2 and COPD [176]. Lp-PLA2 is involved in inflammatory processes that contribute to the development of COPD. It is associated with the activation of inflammatory factors, oxidative stress, and NF-κB signaling pathways, which play a key role in COPD progression. In this regard, Lp-PLA2 has been recognized as a promising biomarker of COPD. Lp-PLA2 levels correlate with various clinical parameters such as FEV1/FVC ratio, BMI, and exercise tolerance. The prognostic performance of Lp-PLA2 for assessing exercise tolerance in patients with COPD is particularly high [177]. Lp-PLA2 levels are also associated with sarcopenia (muscle dysfunction) in COPD patients. Elevated Lp-PLA2 levels adversely affect skeletal muscle mass and function. In mouse models, inhibition of Lp-PLA2 with darapladib improved muscle mass and function, indicating a potential therapeutic benefit [178].

Disorders of lipid metabolism in COPD also occur at the cellular level. Among the cells occupying an important place in the pathophysiology of COPD, alveolar macrophages should be noted. The number of these cells increases significantly in the lungs in COPD. As noted earlier, lipid metabolism plays an important role in the function of alveolar macrophages. Cellular accumulation of cholesterol in macrophages contributes to their pro-inflammatory activation, as well as a decrease in phagocytic activity. Cholesterol accumulation is associated with several known mechanisms, including reduced cholesterol efflux due to decreased functional activity of ABCA1 in smoking [171]. 

COPD is also characterized by abnormalities in the composition of free fatty acids in lung tissue and sputum, highlighting the contribution of free fatty acids to the mechanisms of COPD development [179]. Interestingly, a decrease in plasma free fatty acids is observed in patients with COPD in the early stages [180].

Prostaglandins, particularly PGE2, play a significant role in the pathogenesis of COPD [181]. Serum PGE2 levels are significantly elevated in COPD patients compared to healthy control subjects [182,183]. Higher COX-2 expression and higher concentrations of PGE2 and MMP-2 were found in smokers and COPD patients than in non-smoking control subjects. High levels of PGE2 and MMP-2 correlated with decreased FEV1% values in COPD patients [184]. On the other hand, in COPD, PGE2 levels in induced sputum were associated with a higher frequency of exacerbations and severity of respiratory symptoms [185]. PGE2 levels in exhaled air do not change significantly during infectious exacerbations of COPD, in contrast to other eicosanoids and markers of oxidative stress [186]. PGE2 and its receptors (EP1-4) are involved in the regulation of homeostasis and pulmonary vascular remodeling [187]. PGE2 has previously been shown to reduce pulmonary hypertension in COPD, due to which it and prostacyclin were considered as therapeutic agents for the treatment of pulmonary hypertension [188,189].

Fibroblasts in COPD produce significantly more PGE2 when stimulated by inflammatory cytokines such as IL-1β and TNF-α. This overproduction is associated with increased COX-2 expression and decreased miR-146a levels, leading to chronic inflammation [190]. PGE2 induces senescence of lung fibroblasts, which is characterized by the secretion of inflammatory mediators. This process involves EP2 and EP4 receptor activation, cyclooxygenase-2-dependent reactive oxygen species production, and p53 activation, creating a closed cycle of inflammation and cellular senescence [191].

COPD is characterized by impaired lipoxin production. A decrease in LXA4 concentration in induced sputum has been shown in COPD patients compared to healthy individuals [192,193]. Decreased LXA4 was also determined in exhaled air condensate in patients with moderate to severe COPD [194]. This may be one of the reasons for persistence of inflammation in the airways. It was shown, for example, that in COPD patients in the late phase of exacerbation there was a decreased LXA4 production and increased LTB4/LXA4 ratio in induced sputum, which indicates a pro-inflammatory imbalance [192].

In addition to decreased lipoxins, COPD patients have decreased levels of the lipoxin receptor, which may contribute to the persistence of inflammation in COPD. On the other hand, asymptomatic smokers were found to have increased levels of formyl peptide receptor 1 (FPRL1) receptor in the alveolar walls through which lipoxins act, which may be an adaptive anti-inflammatory mechanism. The number of cells with FPRL1 has also been shown to correlate with the severity of airflow obstruction (FEV1%) in smokers [192,193].

LXA4 reduces inflammation and ferroptosis in cigarette smoke-induced chronic obstructive pulmonary disease via the ALX/FPR2 receptor. In addition, LXA4 intervention reversed the decline in lung function, emphysema, and airway inflammation in COPD mice [195]. In this regard, LXA4 may be a promising candidate for the treatment of COPD.

Interestingly, a recent study showed increased levels of both pro-inflammatory mediators and lipoxins in patients with stable COPD. Moreover, patients with COPD exacerbations had lower levels of D-resolvins than patients with stable COPD and were associated with the risk of severe exacerbations in the future [196]. In this regard, new studies that assess the clinically heterogeneous course of COPD are needed.

Ceramide formation is another important mechanism of lipid metabolism disturbance in the pathogenesis of COPD [197,198]. Ceramides are members of the sphingolipid family and consist of sphingosine and various fatty acids, with the chain length of the fatty acid affecting the physical properties of the ceramide. Ceramides containing fatty acids with 16–24 carbon atoms are most often included in mammalian cell membranes, where they participate in the structural organization of the lipid bilayer [199,200,201]. Due to their biophysical properties, ceramide molecules can self-associate, creating special microdomains that have unique biophysical properties, as they have high structural rigidity, mechanical stability, and compactness of lipid bilayers. Therefore, ceramide-enriched microdomains may be platforms for some signaling pathways [202,203]. Ceramides can be formed by the action of acid sphingomyelinase on the exoplasmic sheet of the plasma membrane. Ceramides can act as a signaling molecule for apoptosis and are associated with the development of many diseases including diabetes mellitus, cardiovascular disease, and non-alcoholic fatty liver disease (NAFLD) [204,205]. Increased ceramide content in the systemic bloodstream correlates with plasma activation of acidic sphingomyelinase characteristic of smokers [206,207]. Ceramide-14 (Cer-14) and Cer-18 ceramides and sphingomyelins SM-14, SM-16, and SM-18 were shown to be associated with a higher likelihood of developing COPD, as Cer-18 and sphingomyelin-18 (SM-18) were associated with lower FEV1 values after adjustment for multiple comparisons [208].

Ceramides may act as one of the mechanisms of clinical heterogeneity of COPD, for example, they participate in the development of emphysema. One of the main mechanisms of emphysema is impaired vascularization of interalveolar septa, which is associated with apoptosis of endothelial cells and alveolar epithelial cells [209]. In this regard, ceramide may be involved in the pathogenesis of emphysema through participation in apoptosis [197,198,210]. Plasma levels of sphingomyelins have been shown to be associated with emphysema, whereas glycosphingolipids are associated with COPD exacerbations [211]. Ceramides may also contribute to lower extremity muscle wasting and physical weakness, another important extrapulmonary manifestation of COPD. One mechanism may be ceramide suppression of amino acid transport and protein synthesis in skeletal muscle cells [212].

Thus, COPD is characterized by various disorders of lipid metabolism, both in the lungs and in the body as a whole. Influencing the disturbed pathways of lipid metabolism is considered as an important potential therapeutic target. It should be taken into account that COPD is a clinically heterogeneous disease that includes both pulmonary and extrapulmonary clinical manifestations.

### 3.2. Clinical Significance of Lipid Metabolism Disorders in Asthma

Asthma is one of the most important chronic respiratory obstructive diseases. Uncontrolled asthma is associated with reduced quality of life, disability, hospitalizations, and economic and social burden. Chronic inflammation in the airways plays an important role in the pathogenesis of asthma and is an area of intense research to improve the diagnosis and therapy of this disease.

Disorders of lipid metabolism play an important role in the pathogenesis of asthma. Dyslipidemia has been associated with severe asthma, with an increased incidence of severe adverse events and moderate to severe adverse events during 12 months of follow-up [213]. It is shown that LDL level is a significant risk factor for the development of chronic obstructive pulmonary disease [214].

Of particular interest in asthma is the involvement of lipid mediators of inflammation. As already mentioned, pro-inflammatory eicosanoids are formed from arachidonic acid (AA) by two enzymatic pathways (Figure 2). The cyclooxygenase (COX) pathway produces prostaglandins and thromboxane.

PGD2 is abundant in the airways in asthma, causing allergic inflammation [110,215]. PGD2 is produced by mast cells and Th2 cells and binds to DP1/DP2 receptors, promoting eosinophil recruitment, Th2 polarization, and bronchospasm [216,217,218,219].

PGE2 has the dual function of activating EP2/EP4 receptors to relax the airways, but in chronic conditions it increases inflammation with EP3. PGE2 activates EP2 and EP4 receptors that are expressed in human airway smooth muscle (HASM). Activation of these receptors results in bronchodilation and inhibition of HASM proliferation, promoting airway relaxation [220,221]. This bronchodilating effect is useful in the treatment of asthma as it helps to prevent bronchospasm and maintain airway patency [220]. EP4 receptor activation also helps to suppress inflammatory responses by inhibiting the activation of innate lymphoid cells group 2 (ILC2) and reducing cytokine production [222,223]. In chronic cases, PGE2 can increase inflammation through the EP3 receptor. Studies have shown that deletion of the EP3 receptor in mice leads to increased inflammation, suggesting that EP3 agonists may lead to a new approach to asthma treatment [220]. Activation of this receptor may enhance inflammatory responses, contributing to the chronicity of asthma symptoms.

Activation of the EP4 receptor by PGE2 can also lead to increased mucus production by goblet cells, which is a characteristic feature of severe asthma. This effect is mediated by increased levels of *MUC5AC* mRNA and protein, which promotes mucus hypersecretion and airway obstruction [224].

Thromboxane A2 (TXA2) induces bronchospasm and smooth muscle proliferation via TP receptors. The bronchoconstrictor response to TXA_2_ is highly dependent on vagal innervation and is sensitive to muscarinic acetylcholine receptor antagonists, indicating a complex interaction between TP receptors and the nervous system [225,226]. TXA_2_ promotes smooth muscle cell proliferation in various tissues, including pulmonary artery smooth muscle cells (PASMCs) and bronchial smooth muscle cells (BSMCs) [227,228]. TP receptor antagonists and TXA_2_ synthase inhibitors such as ozagrel have shown efficacy in reducing smooth muscle proliferation and attenuating bronchospasm, making them promising for the treatment of diseases such as asthma and pulmonary hypertension [227,229].

Leukotrienes (LTs) are formed in the lipoxygenase (LOX) pathway. Cysteinyl LTs (CysLTs: LTC_4_, LTD_4_, LTE_4_) bind to CysLT1/2 receptors, causing severe bronchoconstriction, mucus secretion, and attraction of eosinophils. CysLTs are known to cause the severe bronchoconstriction that is a hallmark of asthma. This effect is mediated by activation of CysLT1R and CysLT2R, which leads to contraction of airway smooth muscles [230,231]. CysLTs also stimulate mucus secretion, contributing to airway obstruction and impaired mucociliary clearance, which plays an important role in asthma [232,233]. CysLTs increase endothelial cell permeability, leading to plasma exudation and edema [234].

Leukotriene receptor antagonists, such as montelukast and zafirlukast, are approved in asthma guidelines as an alternative treatment option. Although inferior in efficacy to inhaled glucocorticosteroids, they suppress nasal and bronchial mucosal inflammation provoked by allergen and non-specific triggers, have marked anti-allergic and anti-inflammatory effects, and are effective both in suppressing symptoms and for the prevention of symptoms of asthma and allergic rhinitis. On the other hand, there are reports that these drugs are characterized by neuropsychiatric adverse events, causing agitation, anxiety, and depression [235,236].

Leukotriene B_4_ (LTB_4_) is a potent pro-inflammatory lipid mediator that plays an important role in attracting and activating neutrophils, contributing to the inflammation seen in severe asthma. The mechanism by which LTB_4_ exerts its effects involves its interaction with specific receptors on neutrophils, primarily BLT1 and BLT2. LTB_4_ acts as a major chemoattractant for neutrophils, directing them to foci of inflammation in the airways [237,238,239]. LTB_4_ signaling through BLT1/BLT2 is associated with NLRP3 inflammasome activation and IL-1β synthesis, which play a key role in neutrophil-dominated airway inflammation [238]. Potential therapeutic strategies are directed towards the development of BLT1 and BLT2 receptor antagonists, 5-lipoxygenase inhibitors, which have shown promising results in preclinical studies [240,241,242].

Arachidonic acid is converted to 15-hydroxyeicosatetraenoic acid (15-HETE) in the 15-lipoxygenase (15-LOX) pathway. Elevated levels of 15-HETE are associated with more severe asthma. The expression of 15-LOX and its product 15-HETE is higher in patients with asthma, which correlates with disease severity and *MUC5AC* expression [243]. Aspirin-sensitive patients with asthma show increased production of 15-HETE upon exposure to aspirin, which is not observed in patients with normal response to aspirin. This suggests a specific pathway involving 15-LOX in aspirin-induced asthma attacks [244,245].

It should be noted that 15-HETE can act as a pro-inflammatory and anti-inflammatory mediator. It is involved in the acute inflammatory response in asthma and its dysregulation may lead to chronic inflammation [246]. Measurement of 15-HETE levels, especially in response to aspirin administration, may serve as a diagnostic tool to identify aspirin-sensitive patients with asthma. Aspirin-induced 15-HETE production in peripheral blood leukocytes is a specific and sensitive test for the identification of aspirin-sensitive patients (ASPITest) [244].

SPMs, which are derived from omega-3/6 fatty acids (Figure 2), are of particular interest in asthma. Lipoxins counteract the pro-inflammatory action of leukotrienes and promote resolution of inflammation. Lipoxins are produced during asthma and help maintain airway homeostasis by blocking asthmatic responses and reducing airway hyperresponsiveness [247]. Lipoxin biosynthesis and lipoxin receptor expression in the airways are reduced in severe asthma [248,249,250]. In severe asthma, including aspirin intolerance and steroid-dependent asthma, liproxin production is often impaired, which contributes to the persistence of airway inflammation and hyperresponsiveness [247,250,251]. 

LXA4 can modulate LTC4-induced airway obstruction and act as an endogenous leukotriene sulfidopeptide receptor antagonist [252]. Severe asthma is characterized by impaired signaling mechanisms regulating lipoxin. LXA4 levels in the airways and the expression of enzymes and receptors involved in lipoxin biosynthesis have been found to be significantly reduced in severe asthma [250]. In another study, in activated whole blood from patients with severe asthma, mean LXA4 levels were lower compared to moderate asthma, in contrast to pro-inflammatory cysteinyl leukotrienes, which were elevated in samples from patients with severe asthma [249]. Lipoxin receptor agonists such as BML-111 have been shown to suppress key inflammatory processes (e.g., TLR2/MyD88/NF-κB) and reduce cytokine production in a mouse model, further supporting their therapeutic potential in asthma [253].

Resolvins (Rvs) represent a promising treatment for asthma due to their potent anti-inflammatory and resolving properties. Resolvins, particularly resolvin E1 (RvE1) and resolvin D1 (RvD1), have been shown to suppress airway inflammation and hyperresponsiveness in models of asthma. RvE1 reduces eosinophil and lymphocyte counts, Th2 cytokine levels, and airway hyperresponsiveness when administered during both sensitization and provocation phases in mouse models [254,255]. RvD1 was found to suppress the production of pro-inflammatory chemokines such as IL-8 and oxidative stress markers such as hydrogen peroxide in bronchial epithelial cells, indicating its ability to reduce inflammation induced by external factors such as cigarette smoke [256].

Platelet-activating factor (PAF) is a phospholipid mediator involved in the pathogenesis of asthma through various mechanisms. PAF is produced by several inflammatory cells including endothelial cells, macrophages, neutrophils, eosinophils, monocytes, and mast cells. It activates these cells, contributing to the inflammatory response in asthma [257,258]. PAF enhances eotaxin production and vascular cell adhesion molecule (VCAM)-1 expression in human lung fibroblasts, promoting the recruitment of eosinophils to the airways in the presence of IL-4 [259]. PAF increases vascular permeability, which is mediated by secondary lipid mediators such as PGE2 and ceramide [260]. Clinically, PAF can cause bronchoconstriction and bronchial hyperresponsiveness and impair gas exchange, mimicking some of the abnormalities seen in asthma [258,260]. Despite their theoretical benefit, PAF receptor antagonists have not shown clear clinical benefits in the treatment of asthma [258,261].

Sphingolipids play an important role in the pathogenesis of asthma. Sphingosine-1-phosphate (S1P) is a bioactive metabolite of sphingolipids that plays an important role in the pathogenesis of asthma. S1P is critical for immune cell trafficking and is elevated in the lungs of patients with asthma. It regulates pulmonary epithelial permeability and mast cell responses, contributing to allergic inflammation and airway hyperresponsiveness [262,263]. S1P promotes mast cell degranulation, resulting in the release of inflammatory mediators that exacerbate asthma symptoms. Sphingosine kinase 1 (SPHK1) is particularly important in this process [264,265]. S1P increases the production of cytokines such as IL-4, IL-13, and IL-17, which are associated with the inflammatory response in asthma [266,267]. S1P promotes airway remodeling by inducing phenotypic changes in lung fibroblasts and stimulating proliferation and contraction of airway smooth muscle. This leads to increased airway hyperresponsiveness and obstruction [268,269,270].

Sphingosine kinase inhibitors such as PF543 and SK1-I have shown promising results in reducing airway inflammation, goblet cell metaplasia, and airway hyperresponsiveness in animal models. These inhibitors affect the sphingosine kinase isoenzyme SPHK1, which plays a key role in S1P production [264,265]. Targeting S1P receptors (S1PR1–S1PR5) is another therapeutic target as it can modulate immune cell function and reduce inflammation. For example, the S1P analog FTY720 has been shown to suppress airway remodeling and hyperresponsiveness in models of asthma [271,272]. In a mouse model, it has been shown that combining S1P pathway inhibitors with other therapies such as disodium cromoglycate (DSCG) can enhance the therapeutic effect by regulating mast cell activity and reducing IgE-dependent responses [273].

Ceramides are also involved in the pathogenesis of asthma. Ceramide levels increase after allergen exposure and apoptosis, reactive oxygen species formation, and neutrophil infiltration, which are characteristic of the severe asthmatic phenotype, as well as the formation of Creola bodies found in the sputum of patients with severe asthma. In this regard, ceramide levels can be used as a biomarker of disease severity [274].

Increased ceramide levels in allergic asthma are associated mainly due to increased degradation of sphingolipids [274,275]. ORMDL proteins such as ORMDL3 play an important role in sphingolipid homeostasis and synthesis [276]. These proteins inhibit serine palmitoyl-CoA transferase (SPT), an enzyme that limits the rate of de novo sphingolipid synthesis. As a result, this protein negatively affects sphingolipid formation, whose decreased synthesis increases bronchial reactivity in the absence of inflammation [277]. Conversely, increased sphingolipid synthesis decreases airway hyperresponsiveness. The glycine hydrazide GlyH-101 (which is a selective and reversible blocker of cystic fibrosis transmembrane conductance regulator (CFTR) chloride channels) and synthetic retinoid fenretinide have been shown to increase sphinganine and dihydroceramide levels in lung epithelial and airway smooth muscle cells, decrease intracellular calcium concentration in airway smooth muscle cells, and reduce agonist-induced contraction of proximal and peripheral airways [278]. The role of the sphingolipid mediators S1P and ceramide as important signaling molecules involved in airway hyperresponsiveness, mast cell activation, and inflammation has been demonstrated in models of allergic asthma [279]. Ceramide/S1P imbalances potentially associated with *ORMDL3* and *SGMS1* gene polymorphisms have been observed in patients with uncontrolled asthma. *ORMDL3* polymorphisms are associated with an increased risk of asthma [280]. Thus, ceramide/S1P synthesis can be used to control airway inflammation in asthma.

Thus, disorders of lipid metabolism play an important role in the pathogenesis of asthma and are therefore a promising therapeutic target. With a good theoretical basis and the results of preclinical studies, the clinical use of drugs acting on disturbed lipid mechanisms in asthma is still a promising target.

### 3.3. Clinical Significance of Lipid Metabolism Disorders in Idiopathic Pulmonary Fibrosis

Idiopathic pulmonary fibrosis (IPF) is a chronic irreversible interstitial lung disease characterized by a progressive decline in lung function. In idiopathic pulmonary fibrosis, abnormalities of lipid metabolism are manifested both at the systemic level (e.g., dyslipidemia) and locally in lung tissue. These abnormalities affect different lipid classes and their metabolic pathways, which contributes to the progression of fibrosis [281]. Lower total cholesterol levels have been identified as an independent risk factor for increased mortality in patients with idiopathic pulmonary fibrosis [282]. High-density lipoprotein cholesterol (HDL-C) levels tend to be lower in patients with IPF compared with controls, and a lower HDL-C/C-reactive protein ratio is associated with a worse prognosis [283].

Epithelial cell dysfunction is a central component of the pathophysiology of IPF. As noted, there are two types of alveolar epithelial cells: type I alveolar epithelial cells (AEC1) and AEC2 cells. AEC2 cells are the most active cells of lung lipid metabolism, including participation in surfactant synthesis. Under conditions of repeated and prolonged AEC2 cell injury, cellular changes including endoplasmic reticulum (ER) stress, apoptosis, and inflammatory and pro-fibrotic signals can occur, stimulating the proliferation and differentiation of lung fibroblasts into highly active myofibroblasts capable of synthesizing extracellular matrix (ECM). Excessive deposition of extracellular matrix leads to deformation and destruction of alveolar structures. Thus, AEC2 cells and fibroblasts are critical regulators of IPF progression [284]. 

Disruption of fatty acid metabolism plays an important role in the pathogenesis of IPF. Changes in fatty acid metabolism include impaired β-oxidation [285]. A balance between fatty acid synthesis and the catabolic process of fatty acid oxidation in mitochondria in AEC2 cells is required for surfactant production [286]. Exposure to cigarette smoke leads to impaired glycolysis in AEC2 cells, which is compensated by increased fatty acid oxidation. Expression of FASN, which is required for fatty acid production, is significantly decreased in the lungs during IPF, leading to mitochondrial dysfunction and increased cell death. Increased expression of FASN has been shown to reduce lung damage and fibrosis in experimental models, indicating its protective role against fibrosis [287].

Disruption of lipid synthesis can lead to an accumulation of proteins synthesized in the endoplasmic reticulum, causing prolonged endoplasmic reticulum stress [288]. Disruption of lipid metabolism leads to mitochondrial dysfunction, impaired regenerative function of epithelial cells, and conversion of fibroblasts into myofibroblasts [284,289]. 

Lysophosphatidic acid (LPA) is synthesized during lung injury and binds to LPA1/LPA2 receptors on fibroblasts, stimulating their proliferation and collagen synthesis. High levels of LPA in bronchoalveolar fluid correlate with fibrosis activity. Lysophosphatidic acid and its receptors have been identified as potential targets because of their role in the development of fibrosis [290]. LPA signals found in various cells, including alveolar epithelial cells, vascular endothelial cells, and fibroblasts, enhance pulmonary fibrosis via LPA receptors by inducing mitochondrial dysfunction, epithelial damage, and transcription of pro-fibrotic cytokines [290].

S1P is involved in fibroblast migration and myofibroblast activation via S1PR2/S1PR3 receptors. Laboratory studies show that S1P promotes lung fibrosis by enhancing the alveolar epithelial-to-mesenchymal transition (EMT) and myofibroblast activation and regulating alveolar endothelial function [291].

Ceramide levels are elevated in the lungs of patients with IPF. This elevation is associated with cell damage and fibrosis, suggesting that ceramides may play a role in the pathogenesis of IPF. Ceramides induce autophagy and apoptosis in lung cells, which may lead to destruction of alveolar epithelial cells and contribute to the fibrotic process [197]. In IPF, ceramide-induced apoptosis and autophagy are not compensated by cell proliferation, leading to tissue damage and fibrosis [197].

3-hydroxy-3-methylglutaryl-CoA synthase 2 (HMGCS2) is involved in the regulation of lipid metabolism of AEC2 cells at the onset and progression of pulmonary fibrosis, making it another target for clinical interventions [292]. Oxidized forms of cholesterol (e.g., 27-hydroxycholesterol) and phospholipids induce cellular damage and enhance the production of TGF-β, a key mediator of fibrosis [107]. Studies have shown that Ox-LDL can induce TGF-β1 production in human alveolar epithelial A549 cells. This process involves the Ras/ERK/PLTP pathway, leading to increased phosphorylation of Smad3 and production of TGF-β1 [293]. This pathway is crucial because TGF-β1 is a major mediator in the pathogenesis of pulmonary fibrosis [294,295].

PGE2 plays an important role in the pathogenesis and potential treatment of IPF due to its anti-fibrotic properties. PGE2 suppresses fibroblast proliferation and collagen synthesis, which are key processes in the development of fibrosis [296,297,298]. PGE2 production depends on the enzyme cyclooxygenase-2 (COX-2), which is inhibited in IPF, resulting in decreased PGE2 levels [297,299]. Inhalation of liposomal PGE2 has shown promising results in reducing fibrosis and improving survival in animal models of IPF [300]. PGE2 disrupts TGFβ signaling and suppresses myofibroblast differentiation, but practical strategies to increase tissue PGE2 levels in IPF are limited [301]. Therapeutic strategies aimed at increasing PGE2 levels or enhancing its signaling pathways may be effective in the treatment of IPF.

LXA4 has demonstrated significant potential in reducing inflammation and fibrosis in IPF. It inhibits proliferation of human lung myofibroblasts (HLMFs), decreases collagen secretion, and reduces alpha smooth muscle actin (α-SMA) expression and Smad2/3 activation, which are key markers of fibrosis. LXA4 can promote regression of myofibroblasts to a resting fibroblast phenotype, thereby attenuating the fibrotic process [302]. The anti-fibrotic action of LXA4 is mediated by its LXA4 receptors, which is expressed in both HLMF in IPF and HLMF in the absence of fibrosis. LXA4 reduces the nuclear translocation of Smad2/3 without inhibiting its phosphorylation, suggesting a specific mechanism for the regulation of fibrosis signaling pathways [302]. In this regard, lipoxins, particularly LXA4, have promising anti-inflammatory and anti-fibrotic properties in IPF.

Thus, disorders of lipid metabolism are characteristic of IPF and are part of its pathogenesis. It should be noted that pulmonary fibrosis has many different mechanisms, a better understanding of which will improve approaches to its diagnosis and treatment.

### 3.4. Clinical Significance of Lipid Mediators in Pulmonary Hypertension

Pulmonary hypertension is a current medical problem and often complicates the course of COPD. The increase in pulmonary artery pressure in these patients ranges from mild to moderate and to the development of severe pulmonary hypertension in some patients. This course of COPD leads to the development of right ventricular heart failure. The cause of pulmonary hypertension in COPD is hypoxic vasoconstriction of pulmonary vessels, leading to their remodeling [303]. 

Interestingly, lipid mediators play an important role in the regulation of pulmonary vascular tone. PGI2 promotes not only bronchial relaxation but also vascular dilation and suppresses platelet aggregation and inflammation [304]. In contrast, TXA2 promotes airway constriction, arterial contraction, and platelet aggregation [120,305]. Smoking contributes to the disruption of the normal balance of prostanoids, leading to a decrease in the PGI2/TXA2 ratio, which contributes to pulmonary vascular remodeling [306].

As in the regulation of inflammation, eicosanoids act multi-directionally via multiple receptors. The major receptors in pulmonary arteries are the prostacyclin receptor (IP), prostaglandin E3 receptor (EP3), and prostaglandin E4 receptor (EP4). Activation of PGD2 receptor 1 (DP1), EP2, EP4, and IP promotes vasodilation and also suppresses proliferation of pulmonary vascular smooth muscle cells (PVSMCs) [120]. On the other hand, EP3 receptors mediate vasoconstriction in human arteries, including in pulmonary hypertension, and are also involved in pulmonary vasoconstriction under the action of isoprostanes, which are elevated in patients with pulmonary hypertension [307,308,309].

Circulating PGI2 and PGE2 levels are decreased in pulmonary hypertension, and IP receptor expression, but not EP4, is reduced. 15-Lipoxygenase (15-LOX) and its metabolite 15-hydroxyeicosatetraenoic acid (15-HETE) have been shown to be upregulated in pulmonary artery cells from patients with pulmonary hypertension as well as in experiments in rats with hypoxia. 15-HETEs promote pulmonary vascular remodeling and the progression of pulmonary hypertension (PH) under hypoxic conditions through stimulation of endothelial cell migration and proliferation of pulmonary artery medial smooth muscle cells. These mechanisms involve action on p38 MAPK signaling pathways, which affects their cell cycle [310,311]. 15-HETE can also constrict pulmonary arteries by increasing intracellular Ca2+ levels and inhibiting voltage-gated K^+^ (Kv) channels. In addition, 15-HETE phosphorylates eNOS, causing a decrease in eNOS activity [312,313]. Thus, 15-HETE produced during hypoxia is an important mediator in the regulation of hypoxic pulmonary hypertension.

On the other hand, specialized pro-resolving lipid mediators may serve as therapeutic targets for the treatment of pulmonary hypertension. It has been shown that RvE1, acting through the ChemR23 receptor, can attenuate experimental pulmonary hypertension in mice by inhibiting Wnt family member 7A (Wnt7a)/β-catenin signaling [314]. In addition, resolvin RvE1 can suppress human pulmonary artery contractility induced by thromboxanes or cytokines. It has also been shown to attenuate injury-induced vascular neointima formation in mice [314,315,316,317].

Thus, a better understanding of the role of lipid mediators in pulmonary function will allow better utilization of their diagnostic capabilities and therapeutic potential.

## 4. Conclusions

Chronic respiratory diseases are an important problem because they are among the most common diseases and also carry an economic and social burden. COPD is one of the leading causes of hospitalizations, disability, and mortality. Despite the apparent simplicity of diagnosis, the disease is often detected late, in the presence of severe obstruction and when the effectiveness of treatment is already insufficient, which reduces the quality of life of patients and increases the likelihood of comorbid diseases and their severity. Asthma is also one of the most important chronic respiratory obstructive diseases. Uncontrolled asthma is associated with reduced quality of life, disability, hospitalizations, and economic and social burden. Chronic inflammation in the airways in COPD and asthma has significant differences but plays an important role in the pathogenesis of both diseases. Lipid metabolism disorders play an important role in the development of COPD and asthma and can be used as biomarkers to diagnose and assess the course of these diseases. Pulmonary fibrosis due to various causes is a serious problem of modern pulmonology. These patients have a progressive decline in pulmonary function and consequently a decrease in quality of life and worsening prognosis.

In this regard, therapeutic approaches aimed at correction of disturbed lipid metabolism in the treatment of lung diseases are of interest (Table 1).

It should be noted that, despite serious theoretical substantiation and numerous results of preclinical studies, the therapeutic potential of correction of lipid metabolism disorders in chronic lung diseases has not been fully realized to date. 

On the other hand, data on how treatment of lipid abnormalities may affect the course of chronic respiratory diseases are also of interest. Statins, known primarily for their cholesterol-lowering properties, have shown potential benefit in patients with COPD. Statins reduce systemic and pulmonary inflammation, decreasing neutrophil infiltration and cytokine production [55,318,319]. Statins are associated with lower rates of COPD exacerbations, hospitalizations, and the need for antibiotic treatment [320,321].

Statins may have not only anti-inflammatory but also anti-fibrotic effects in IPF. In animal models, atorvastatin reduced the level of fibrosis and inflammation markers [322]. In vitro studies have shown that statins inhibit the production of fibrogenic mediators in lung fibroblasts [55,323]. In some studies, the use of statins has been associated with a reduction in all-cause mortality and IPF mortality. For example, one study showed that those who took statins had a lower risk of IPF mortality (OR 0.36; 95% CI 0.14 to 0.95) and lower risk of hospitalization for any cause (OR 0.58; 95% CI 0.35 to 0.94) compared with those who did not take them [324]. Another meta-analysis showed reduced mortality when taking statins (OR 0.89; 95% CI 0.83 to 0.97) for idiopathic pulmonary fibrosis [325].

Thus, lipid metabolism plays an important role in lung function and disorders of lipid metabolism are a promising diagnostic and therapeutic target. Drugs acting on lipid mediators represent a promising tool for the treatment of chronic lung diseases. Further studies of lipid metabolism and signaling pathways will be important for the development of new effective therapies for lung diseases.

## Figures and Tables

**Table 1:** Therapeutic goals in lipid metabolism disorders in patients with chronic lung disease.

Therapeutic Goals	Mechanism	Interventions
Suppression of inflammatory processes	Excess pro-inflammatory lipids (leukotrienes, prostaglandins) increase lung tissue damage	Leukotriene receptor antagonists (montelukast) Inhibitors of eicosanoid synthesis (omega-3 PUFAs)
Reduction of oxidative stress	Lipid peroxidation causes alveolar damage and fibrosis	Antioxidants Stimulation of endogenous antioxidant systems
Correction of cholesterol metabolism disorders	Cholesterol accumulation in macrophages stimulates inflammation through several mechanisms	Statins
Stimulation of anti-inflammatory mechanisms	Specialized pro-resolving mediators promote resolution of inflammation	Specialized pro-resolving mediators and their synthetic analogs

## Data Availability

Not applicable.

## Abbreviations

The following abbreviations are used in this manuscript:

ABCA1	ATP Binding Cassette Subfamily A Member 1
AEC2	alveolar epithelial type II cells
COPD	chronic obstructive pulmonary disease
COX	cyclooxygenase
CSE	cigarette smoke extract
EVALI	e-cigarette and vaping product use-associated lung injury
FASN	fatty acid synthase
FEV1	forced expiratory volume in 1 second
IgE	immunoglobulin E
IL	interleukin
iNOS	inducible nitric oxide synthase
LOX	lipoxygenase
LPCAT1	lysophosphatidylcholine acyltransferase 1
LPS	lipopolysaccharide
LT	leukotriene
LX	lipoxin
LXR	liver X receptor
MaR	maresin
PD	protectin
PH	pulmonary hypertension
PG	prostaglandin
PC	phosphatidylcholine
Rv	resolvin
S1P	sphingosine-1-phosphate
SPT	serine palmitoyl-CoA transferase
TLR4	Toll-like receptor 4
TNFα	tumor necrosis factor-alpha
TXA2	thromboxane A2
